# Molecular Biomarker of Drug Resistance Developed From Patient-Derived Organoids Predicts Survival of Colorectal Cancer Patients

**DOI:** 10.3389/fonc.2022.855674

**Published:** 2022-03-29

**Authors:** Lifeng Chen, Bo Tian, Wen Liu, Haitao Liang, Yong You, Weizhen Liu

**Affiliations:** ^1^ Department of Hematology, Union Hospital, Tongji Medical College, Huazhong University of Science and Technology, Wuhan, China; ^2^ Department of Orthopaedic Surgery, Zhongshan Hospital, Fudan University, Shanghai, China; ^3^ Shenzhen Second People’s Hospital (The First Hospital Affiliated to Shenzhen University, Health Science Center), Shenzhen, China; ^4^ Department of Gastrointestinal Surgery, Union Hospital, Tongji Medical College, Huazhong University of Science and Technology, Wuhan, China

**Keywords:** colorectal cancer, organoids, 5-fluorouracil, drug resistance, molecular biomarker, predict, survival

## Abstract

The drug 5-fluorouracil (5-Fu) is the critical composition of colorectal cancer (CRC) treatments. Prognostic and predictive molecular biomarkers for CRC patients (CRCpts) treated with 5-Fu-based chemotherapy can provide assistance for tailoring treatment approach. Here, we established a molecular biomarker of 5-Fu resistance derived from colorectal cancer organoids (CRCOs) for predicting the survival of CRCpts. Forty-one CRCO cultures were generated from 50 CRC tumor tissues after surgery (82%). The following experiments revealed a great diversity in drug sensitivity for 10 μM 5-Fu treatment tested by using organoid size change. Fourteen cases (34.1%) were 5-Fu sensitive and the other 27 (65.9%) were resistant. Then, differentially expressed genes (DEGs) associated with 5-Fu resistance were outputted by transcriptome sequencing. In particular, DEGs were generated in two comparison groups: 1) 5-Fu sensitive and resistant untreated CRCOs; 2) CRCOs before 5-Fu treatment and surviving CRCOs after 5-Fu treatment. Some molecules and most of the pathways that have been reported to be involved in 5-Fu resistance were identified in the current research. By using DEGs correlated with 5-Fu resistance and survival of CRCpts, the gene signature and drug-resistant score model (DRSM) containing five molecules were established in The Cancer Genome Atlas (TCGA)-CRC cohort by least absolute shrinkage and selection operator (LASSO) regression analysis and 5-fold cross-validation. Multivariate analysis revealed that drug-resistant score (DRS) was an independent prognostic factor for overall survival (OS) in CRCpts in TCGA-CRC cohort (*P* < 0.001). Further validation results from four Gene Expression Omnibus (GEO) cohorts elucidated that the DRSM based on five genes related to 5-Fu chemosensitivity and developed from patient-derived organoids can predict survival of CRCpts. Meanwhile, our model could predict the survival of CRCpts in different subgroups. Besides, the difference of molecular pathways, tumor mutational burden (TMB), immune response-related pathways, immune score, stromal score, and immune cell proportion were dissected between DRS-high and DRS-low patients in TCGA-CRC cohort.

## Introduction

Colorectal cancer (CRC) is the fourth most common diagnosed cancer and the second leading cause of cancer death worldwide (1). CRC tumors are highly heterogeneous in their intratumor and intertumor characteristics because of microsatellite instability (MSI), chromosomal instability (CIN), DNA repair defects, aberrant DNA methylation, and other factors. These factors determine how colorectal cancer patients (CRCpts) respond to specific therapy (2). In the era of precision oncology, implicit molecular characterization of the tumor is essential in defining the best therapeutic plan. Therefore, the establishment of prognostic and predictive molecular biomarkers is increasingly becoming more valuable in cancer treatment (3, 4).

In clinical practice, although new options have been developed including targeted therapy and immunotherapy, chemotherapy based on 5-fluorouracil (5-Fu) is still the critical composition of CRC treatments (5). However, drug resistance is ubiquitous, resulting in tumor progression and poor outcome in CRCpts. For instance, despite advances in response rate with the advent of various modulation strategies such as monoclonal antibodies combined with chemotherapy, 5-year relative survival rate for metastatic colorectal cancer (mCRC) is only slightly over 12% (6). Approximately half of metastatic CRCs are resistant to 5-Fu-based chemotherapies (7). One of the major culprits for this observation is the appearance of drug resistance. Prognostic and predictive molecular biomarkers for CRCpts receiving 5-Fu-based chemotherapy can provide assistance for tailoring treatment approach.

Organoid is a self-organized three-dimensional (3D) construct and constituted of various cell types that ultimately generated from stem cells. It is capable of mimicking the architecture and functionality of primary organs (8). Patient-derived tumor organoids (PDTOs) have been proven to recapitulate the tumor’s pathological morphology, marker expression, chromosomal stability, genomic characterization, and tumor heterogeneity (8, 9). Recently, several studies suggested that PDTOs can predict the response to chemotherapy, chemoradiation, and targeted therapy, suggesting that PDTOs may represent a companion preclinical tool in precision oncology (10–12). However, the success rate of establishing PDTOs from CRCpts still needs to be improved (<90%), and PDTO-based drug assays require at least 1–2 weeks (10–13). These challenges may hamper the implementation of PDTO approach in a clinical setting.

PDTOs can more faithfully represent patient tumors than cell lines that potentially enable more comprehensive insights into mechanisms of drug resistance (8, 9). In this research, we successfully generated a gene signature and score system as molecular biomarkers that can predict the prognosis of CRCpts by using drug sensitivity data (5-Fu) of colorectal cancer organoids (CRCOs). Our model may be helpful in tailoring therapeutic regimens and act as a supplement of PDTO-guided personalized treatment for CRCpts.

## Materials and Methods

### Study Design

#### Study Objectives

To generate a gene signature of chemosensitivity developed from PDTOs and investigate the potential of the gene signature to predict the survival of CRCpts.

#### Research Subjects

Surgical specimens from CRCpts were used to establish a biobank of CRCOs. CRC datasets from The Cancer Genome Atlas (TCGA) program and Gene Expression Omnibus (GEO) database were employed to develop and validate the gene signatures for predicting the survival of CRCpts, respectively.

#### Study Design

The drug sensitivity of CRCOs to 5-Fu were tested, and differentially expressed genes (DEGs) related to 5-Fu resistance were generated by transcriptome sequencing. Gene signature and drug-resistant score model (DRSM) for predicting the survival of CRCpts were developed and validated in TCGA and GEO datasets by using drug-resistant genes (DRGs) associated with 5-Fu resistance, respectively.

**Figure 1:**
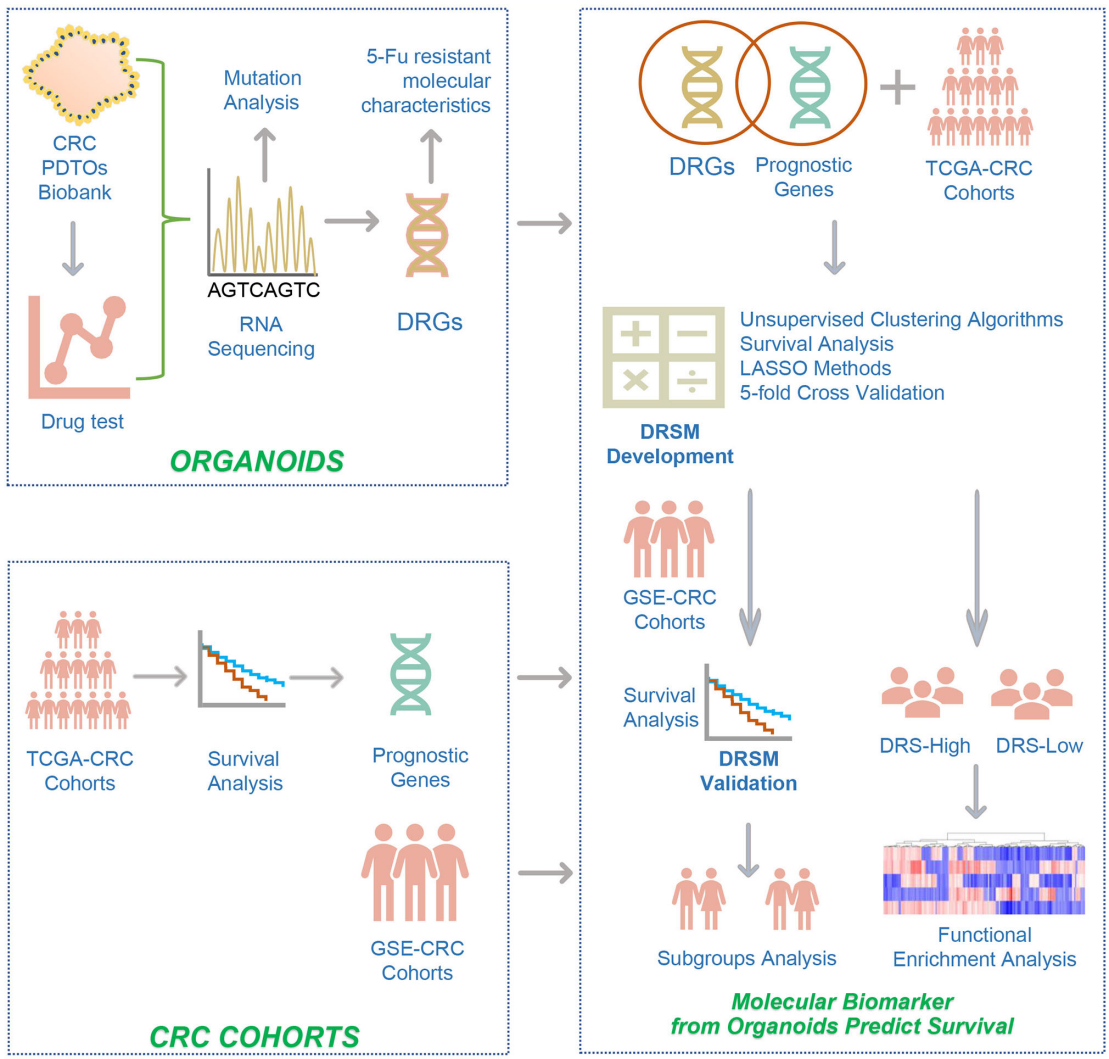
The overall flowchart depicting the development and validation of the gene signatures and drug-resistant score model. The study design was described in detail in the *Methods* (Study design). CRC, colorectal cancer; PDTOs, patient-derived tumor organoids; 5-Fu, 5-fluorouracil; DRGs, drug-resistant genes; TCGA, the cancer genome atlas; GSE, gene expression omnibus series; LASSO, least absolute shrinkage and selection operator; DRSM, drug-resistant score model; DRS, drug resistant score.

The overall flowchart depicting the development and validation of the gene signatures and DRSM was presented in **Figure 1**.

### Tumor Samples of Colorectal Cancer Patients

Fifty surgically resected cancer tissues from previously untreated CRCpts were collected in the Department of Gastrointestinal Surgery, Union Hospital, Tongji Medical College, Huazhong University of Science and Technology. The diameters of the CRC tissues for CRCO culture were about 5.00–10.00 mm. The tissue collections and experiments were reviewed and approved by the institutional review boards of Union Hospital, Tongji Medical College. Informed consents were obtained from all the patients enrolled in this study.

### Processing of Surgical Specimen Tissues

After being harvested, the CRC tissues were transferred into 15-ml centrifuge tubes with cold phosphate buffer saline (PBS) containing gentamicin/amphotericin B (GIBCO, R01510) and normocin (InvivoGen, antnr-1). The tissues were maintained on ice prior to tissue disaggregation and organoid culture.

### The Isolation and Primary Culture of Colorectal Cancer Tissues

Primary cancer cells were isolated and cultured using previously described methods (10, 11, 14). Briefly, CRC tissues were washed in cold PBS containing streptomycin/penicillin (GIBCO, 15140-122) for 5 cycles (5 min per cycle), minced into small pieces, and incubated at 37°C in digestion solution with 10 ml Dulbecco’s Modified Eagle Medium (DMEM) (GIBCO, C1199500BT) containing 1.5 mg/ml collagenase II (Solarbio, C8150), 500 U/ml collagenase IV (Sigma-Aldrich, C9407), 0.1 mg/ml dispase type II (Sigma-Aldrich, D4693), 20 mg/ml hyaluronidase (Solarbio, h8030), 10 mM RHOK inhibitor ly27632 (Sigma-Aldrich, Y0503), and 1% fetal bovine serum. Tumor tissues were resuspended every 5 min. For all cases, the digestion was terminated by adding 10 ml cold PBS until no tissue fragments were left. The suspension was filtered with 100 μm cell strainer, and the tumor cells were collected after centrifugation for 5 min at 300–400g.

Finally, the tumor cells were mixed with Matrigel (Corning, 356231) and seeded into a prewarmed 24-multiwell plate. After the Matrigel has solidified, the tumor cells were cultured in CRCO culture medium containing 1× Advanced DMEM/F12 (GIBCO, 12634-010), 1× 4-(2-hydroxyethyl)-1-piperazineethanesulfonic acid (HEPES) (GIBCO, 15630080), 1× Glutamax (GIBCO, 35050061), 1× Normocin (InvivoGen, ant-nr-1), 1× Gentamicin/amphotericin B (GIBCO, R01510), 1× N2 supplement (Invitrogen, 17502-048), 1× B27 supplement (Invitrogen, 17504-044), 500 ng/ml R-spondin 1 (Sino Biological Inc., 11083-HNAS), 100 ng/ml Noggin (Sino Biological Inc., 50688-M02H), 50 ng/ml epidermal growth factor (EGF) (Sino Biological Inc., 50482-MNCH), 1 mM n-Acetylcysteine (Sigma-Aldrich, A9165), 10 mM Niacinamide (Sigma-Aldrich, N0636), 500 nM A8301 (Tocris, 2939), 3 μM SB202190 (Sigma-Aldrich, S7067), 10 nM Gastrin (Sigma-Aldrich, G9145), and 10 nM Prostaglandin E2 (Sigma-Aldrich, P6532) at 37°C, 5% CO_2_ incubator.

### Colorectal Cancer Organoid Culture

CRCOs were cultured using previously described methods (10, 11, 14). The culture medium of CRCOs was refreshed every 3 days. CRCOs were subcultured every 3–14 days depending on the growth rate of organoids. CRCOs were passaged by mechanical dissociation into small fragments through shearing with 1% Bovine Serum Albumin (BSA)-coated glass pipette tip. For those dense organoids, they were resuspended in prewarmed TrypLE™ Express enzyme (1×) (GIBCO, 12605-010) before mechanical dissociation. After dissociation, CRCOs were washed with cold PBS several times to clear out the Matrigel. Finally, CRCO fragments were resuspended in fresh Matrigel, seeded into a prewarmed 24-multiwell plate, and cultured as described above.

For CRCO cryopreservation, organoids were harvested and mechanically dissociated into small fragments as described above. Then, organoid fragments were mixed with freezing medium (CELLBANKER™ 2, ZENOAQ, 170905) and frozen following standard procedures. As required, the frozen CRCOs were thawed according to standard procedures and cultured as mentioned before. The culture medium was supplemented with 10 μM RHOK inhibitor Y-27632 for the first 3 days of culture after thawing.

### Drug Sensitivity Test of Colorectal Cancer Organoids

The assays for drug sensitivity of CRCOs were conducted as described previously (11). Organoid size change at day 24 to day 0 after treatment was used as the indicator for the judgment of drug sensitivity of CRCOs. The optimal validated cutoff value of organoid size change was 36.42% (11).

Briefly, well-grown CRCOs were mechanically dissociated into small fragments, resuspended in 100% Matrigel (≈10 fragments/μl), seeded into 48-well cell culture plate (15 μl, ≈150 fragments/well), and cultured with 300 μl CRCO culture medium. When organoid size reached about 100 μm (day 0), the culture medium was replaced with 300 μl fresh medium containing 10 μM 5-Fu (Selleck, S1209). After 3 days, the 5-Fu-containing medium was refreshed again. Subsequently, the culture medium was replaced by fresh drug-free CRCO culture medium every 3 days in most cases. The medium was refreshed every 1–2 days during the period from day 7 to day 24 for some cases, which have grown much faster than others.

Images of CRCOs were obtained every 3 days after 5-Fu treatment using a ZEISS microscope (ZEISS, Vert.A1). Then, CRCO size was evaluated by using Image-Pro Plus 6.0 (Media Cybernetics, Inc.) software. About 100 organoids were measured per case.

### RNA Extraction and Preparation

CRCOs in good condition were collected, homogenized in TRIzol™ Reagent (Invitrogen, 15596026), and frozen at -80°C. Organoid RNA was extracted according to the TRIzol Reagent protocol. RNA contamination and degradation were monitored on 1% agarose gels. RNA integrity was evaluated using the RNA Nano 6000 Assay Kit of the Bioanalyzer 2100 system (Agilent Technologies, CA, USA). RNA purity was examined using the NanoPhotometer^®^ spectrophotometer (IMPLEN, CA, USA).

### Transcriptome Sequencing of Colorectal Cancer Organoids

A total amount of 1 µg RNA per sample was used as input material for the RNA sample preparations. Sequencing libraries were generated using NEBNext^®^ Ultra™ RNA Library Prep Kit for Illumina^®^ (NEB, USA) following the manufacturer’s recommendations, and index codes were added to attribute sequences to each sample. Detailed information about library preparation for transcriptome sequencing was attached in **Supplementary Methods**. The clustering of the index-coded samples was performed on a cBot Cluster Generation System using TruSeq PE Cluster Kit v3-cBot-HS (Illumina) according to the manufacturer’s instructions. After cluster generation, the library preparations were sequenced on an Illumina Novaseq platform, and 150-bp paired-end reads were generated.

### Gene Expression and Functional Enrichment Analysis

Fragments per kilobase million (FPKM) was used to evaluate expression levels of individual genes. To identify differentially expressed genes (DEGs), the R package limma was used (15), which implements an empirical Bayesian approach to estimate gene expression changes. DEGs were determined by significance criteria (*P* value <0.05) as implemented in the R package limma (15). The Venn diagram was used to visualize common significant DEGs between the different conditions.

The clusterProfiler (16) R package was performed to demonstrate functional enrichment analysis. We identified functional pathways that were upregulated and downregulated by running a gene set enrichment analysis (GSEA) (17) of the adjusted expression data for all transcripts. Enrichment *P* values were based on 1,000 permutations and subsequently adjusted for multiple testing using the Benjamini–Hochberg procedure to control the false discovery rate (FDR). A developing R package enrichplot (https://github.com/GuangchuangYu/enrichplot) implements several visualization methods to help interpret enrichment results and was adopted to visualize GSEA results.

### Datasets of Colorectal Cancer in The Cancer Genome Atlas and Gene Expression Omnibus Databases

CRC datasets from TCGA and GEO databases were used for the development and validation of gene signature and DRSM for predicting the survival of CRCpts, respectively. It was worth noting that only stage II–IV CRCpts were enrolled in the current study because patients with stage I disease underwent surgical resection but did not receive 5-Fu chemotherapy.

#### The Cancer Genome Atlas Datasets

The clinical and gene expression data (FPKM, fragments per kilobase of exon model per million reads mapped) of CRCpts (TCGA-COAD and TCGA-READ) were obtained from the Genomic Data Commons (GDC) Data Portal (https://portal.gdc.cancer.gov/) by using TCGAbiolinks (18).

#### Gene Expression Omnibus Datasets

The CRC datasets were preliminarily screened by using the search query as follows: (“colorectal neoplasms”[MeSH Terms] OR colorectal cancer[All Fields]) AND “Homo sapiens”[porgn] AND ((“gds”[Filter] OR “gse”[Filter]) AND (“Expression profiling by array”[Filter] OR “Expression profiling by high throughput sequencing”[Filter]) AND (“50”[n_samples]: “10000”[n_samples])) in GEO database. The datasets derived from cell lines and other irrelevant datasets were eliminated manually. In particular, CRC datasets were also enrolled through literature review to avoid missing valuable datasets. Then, the datasets were obtained by using GEOquery. The preliminarily selected GEO datasets were as follows: GSE40967, GSE17538, GSE87211, GSE24551, GSE38832, GSE33113, GSE14333, GSE39084, GSE71187, GSE12945, and GSE29623.

### Univariate and Multivariate Survival Analyses

For filtration of the prognosis-related genes, we calculated the prognosis related *P* value of each gene using univariate and multivariate survival analyses. The Kaplan–Meier method was used to generate survival curves, and the log-rank test was used to determine the statistical significance of differences. The hazard ratios for univariate analysis were calculated using the Cox proportional hazards regression model. A multivariate Cox regression model was used to determine independent prognostic factors using R coxph package. Genes with *P* values <0.05 were considered significant.

### Development of the Drug-Resistant Score Model

Then, the least absolute shrinkage and selection operator (LASSO) regression model implemented in the glmnet (v4.0-2) package was used for the next-step filtration of genes. LASSO regression penalizes the data-fitting standard by eliminating predictive variables. To evaluate the variability and reproducibility of the estimates produced by the LASSO regression model, we repeated the regression fitting process and calculated the best lambda to reduce the error rate by 5-fold cross-validation. Twenty-six genes with non-zero coefficient estimates were retained. The multivariate Cox regression model was used to estimate the coefficient and prognosis-related *P* value of each gene. Five genes were identified as significant with *P* value <0.05, for considering as independent prognostic factors. LASSO regression was performed to construct the score model shown as follows: DRS = GEL (gene expression level) (*CACNA1D*) × -0.0563 + GEL (*CIITA*) × -0.0356 + GEL (*PFN2*) × 0.0332 + GEL (*SEZ6L2*) × 0.0378 + GEL (*WDR78*) × -0.0386.

The R package MaxStat (https://CRAN.R-project.org/package=maxstat) was used to test possible cut points and find the one achieving the maximum rank statistic to separate datasets into score-low and score-high groups. R package forestplot was used for presentation of the results of GEO datasets and TCGA dataset.

### Statistical Analysis

The *P* values were two-sided. A value of *P* < 0.05 was considered as statistically significant. CRCO size (day24/d0) was selected as the parameter to evaluate the sensitivity of CRCOs to 5-Fu treatment, and 36.4% was used as the cutoff following the results from previous research (11). Wilcoxon rank-sum test was used for comparison of two groups. Correlation coefficients were computed by Spearman and distance correlation analyses. Two-sided Fisher exact tests were used to analyze contingency tables. To identify significant genes in the differential gene analysis, we applied the Benjamini–Hochberg method to convert the *P* values to FDRs. All heatmaps, including unsupervised hierarchical clustering, were generated by the function of pheatmap (https://github.com/raivokolde/pheatmap). The statistics of survival analysis, RNA sequencing, gene expression, and functional enrichment analysis were specifically described above.

## Results

### Establishment of 41 Colorectal Cancer Organoid Lines

From April 2018 to August 2018, we obtained 50 surgically resected cancer tissues from previously untreated CRCpts. All of them were adenocarcinoma. Cancer cells were isolated and cultured in 3D Matrigel by using the procedures as reported by Hans Clevers group (14, 19). Considering the interpatient tumor heterogeneity, we also specifically referred to the impressive experience from Fujii (20) to improve the success rate of culture. We favorably generated 41 organoid cultures from 50 tumor tissues (82%). For two, we did not observe growth. The complete structures of two were disrupted after several days of swelling. The other five were lost due to bacterial/yeast infection. Additional analysis showed that PTDO generation was not correlated with patients’ characteristics (**Table S1**). It has been well confirmed that CRCOs recapitulated characteristics of CRC primary tumor tissues (10, 11, 14). Note that because normal human colon epithelial organoids require Wnt ligand (Wnt 3a) in the culture medium (19), it was considered that organoids cultured in Wnt3a-free media were CRCOs and further characterization relative to the primary tumor was not undertaken. Therefore, the histopathological and genomic (DNA sequencing) features were not characterized in the current study to confirm that organoids derived from cancer patients can recapitulate the features of corresponding tumors.

### Sensitivity of Colorectal Cancer Organoids to 5-Fluorouracil

**Figure 2:**
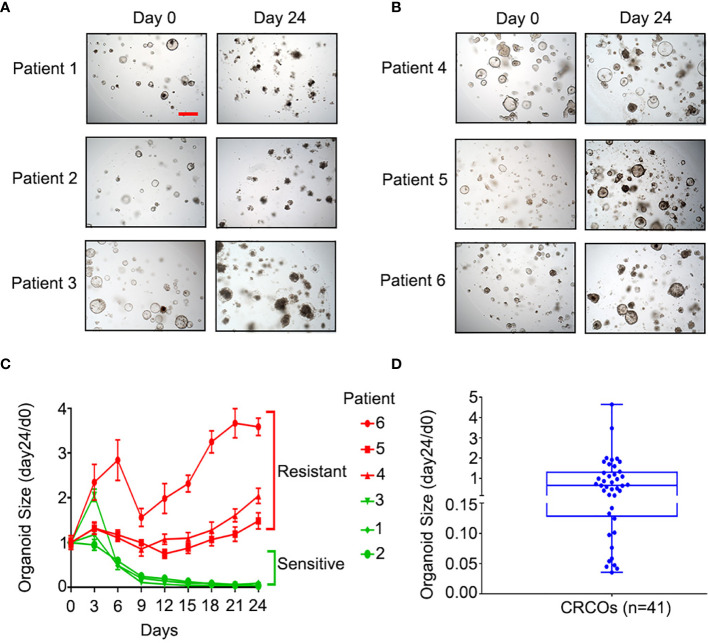
Sensitivity of CRCOs to 5-Fu. **(A)** Representative bright-field images of 5-Fu-sensitive CRCOs at day 0 and day 24 after 10-μM 5-Fu treatment in three selected cases. *The CRCOs with disrupted structures are dead and do not have the ability to repopulate.* **(B)** Representative bright-field images of 5-Fu-resistant CRCOs at day 0 and day 24 after 10-μM 5-Fu treatment in three selected cases. *CRCOs with complete structures are alive and have the ability to repopulate*. Scale bar, 200 μm. **(C)** CRCO size change after 10-μM 5-Fu treatment in six selected cases. The data shown are means with SEM from 8 duplicates. **(D)** Box plot of CRCO size change (day24/d0) in all of the 41 cases. Within the box, the horizontal blue center line denotes the median value (50th percentile), while the box contains the 25th to 75th percentiles of the distribution of values. The blue whiskers mark the minimum and maximum of the values. CRCOs, colorectal cancer organoids; 5-Fu, 5-fluorouracil; SEM, standard error of mean.

*Ex vivo* drug sensitivity screen in 3D cancer organoid culture nominates therapeutic candidates (14, 21). Cell viability testing using ATP detection assay was the most common approach for drug sensitivity evaluation of cancer organoids (10, 14, 22, 23). Organoid size change, serving as a measure of organoid survival, is as effective as CellTiter-Glo 3D cell viability assay (11) and is more economical and easier to use. We tested the sensitivity of 41 CRCO lines to 5-Fu by using this method (**Figures 2A–D**). The kinetic size change curves and ratios of CRCO size at day 24 to day 0 [CRCOs size (day24/d0)] after 5-Fu treatment revealed great diversity in drug sensitivity for 10-μm 5-Fu treatment (**Figures 2C, D** **)**, which is consistent with the widely divergent response of CRCpts to 5-Fu-based chemotherapy (24, 25).

We chose CRCO size change (day24/d0) as the parameter to evaluate the sensitivity of CRCOs to 5-Fu treatment and 36.4% as the cutoff according to a previous study (11). CRCO size change (day24/d0) ranged from 0.035 to 4.65 (**Figure 2D**). Fourteen cases (34.1%) were 5-Fu sensitive and the other 27 (65.9%) were resistant (**Figure 2D**). Additional analysis showed that organoid sensitivity to 5-Fu was not correlated with patients’ characteristics (**Table S1**). After testing the drug sensitivity of CRCOs to 5-Fu, we continually cultured and expanded the surviving organoids that were resistant for transcriptome sequencing analysis subsequently.

### 5-Fluorouracil-Resistant Molecular Characteristics of Colorectal Cancer Organoids

**Figure 3:**
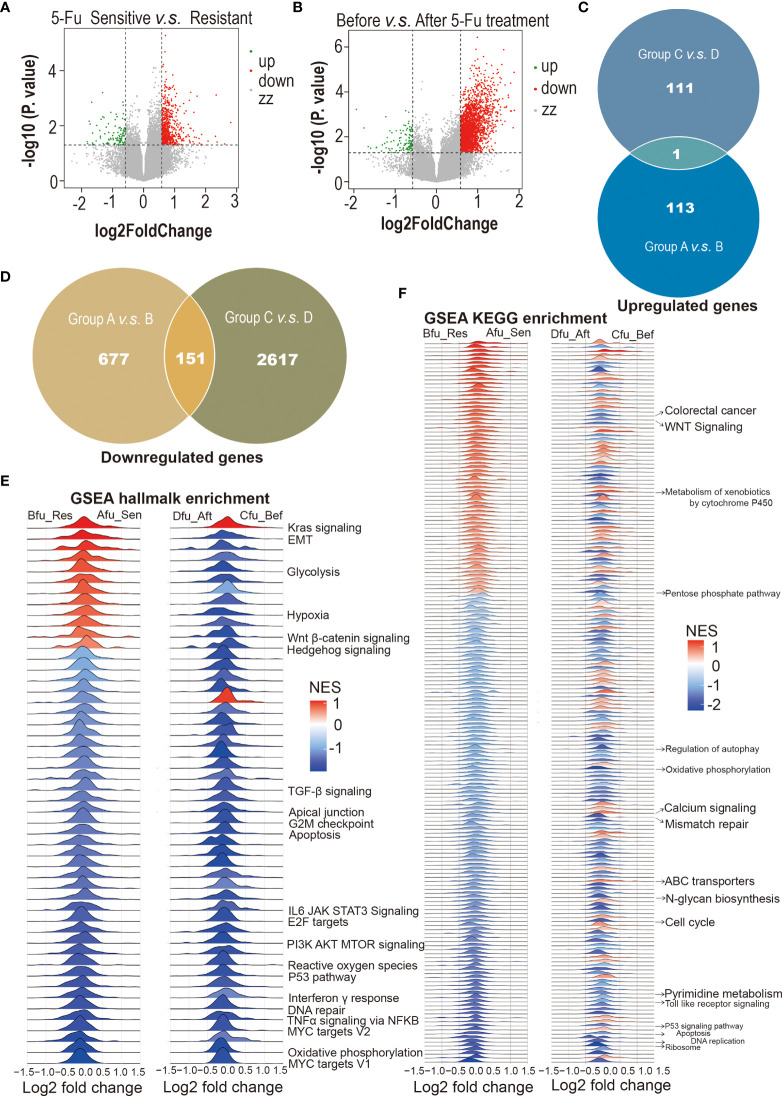
5-Fu-resistant molecular characteristics of colorectal cancer organoids (CRCOs). **(A)** Volcano plot for differentially expressed genes between 5-Fu-sensitive (group A) and -resistant (group B) untreated CRCOs. **(B)** Volcano plot for differentially expressed genes between CRCOs before 5-Fu treatment (group C) and surviving CRCOs after 5-Fu treatment (group D). **(C)** Venn diagram showed that only 1 upregulated gene was overlapped in the two comparison groups. **(D)** Venn diagram showed that 151 downregulated genes were overlapped in the two comparison groups. **(E, F)** Gene set enrichment analysis (GSEA) using the hallmark and KEGG gene sets to dissect the pathways associated with 5-Fu resistance, respectively. The pathways that have been validated in literature were marked. 5-Fu, 5-fluorouracil; GSEA, gene set enrichment analysis; Res, resistant; Sen, sensitive; Aft, after; Bef, before; NES, normalized enrichment score; KEGG, kyoto encyclopedia of genes and genomes; CRCOs, colorectal cancer organoids.

Many intrinsic and extrinsic factors involved in 5-Fu resistance in CRC have been well studied (26, 27). Here, CRCOs were employed for the first time to reveal 5-Fu resistance mechanisms of CRC. We utilized transcriptome sequencing to dissect 5-Fu-resistant molecular characteristics of CRCOs (**Figures 3A, B** **)**. DEGs were generated in two comparison groups: 1) 5-Fu sensitive (*group A*) and resistant (*group B*) untreated CRCOs; 2) CRCOs before 5-Fu treatment (*group C*) and surviving CRCOs after 5-Fu treatment (*group D*) (**Figures 3A, B** **)**. Principal component analysis (PCA) showed a high degree of similarity between groups A/B and C/D, respectively (**Figures S1A, B**). Therefore, the criteria of *P* < 0.05 and logFC > 0.58 (fold change >1.5) were used for identification of DEGs (28). Here, 113 and 111 genes were upregulated, and 677 and 2,617 genes were downregulated in groups B and D compared with groups A and C, respectively (**Figures 3C, D** **)**. Only 1 upregulated and 151 downregulated genes were overlapped in the two comparison groups (**Figures 3C, D** **)**, demonstrating that these two different comparison groups (A vs. B, C vs. D) revealed vastly divergent DEGs. The detailed information of DEGs was listed in **Table S2**.

Some of these DEGs have been reported to participate in regulating 5-Fu resistance in CRC. For instance, several genes upregulated in *Group B* or *Group D*, including *ALDOA* (29), *GLUT2 (SLC2A1)* (30, 31), *NACC1* (31), *POLR2A* (32), and *TGFB1* (33), promote 5-Fu resistance in CRC. Besides, some DEGs (*FERMT1* (34), *HEY2* (35, 36), *ITGB4* (37, 38), *PDXP* (39), *TIMP1* (40), TP53I3 (39), et al.) probably have a role in 5-Fu resistance in CRC. Interestingly, the expression levels of most explored enzymes (*TYMS*, *MTHFR*, *TP*, et al.) involved in the resistance of 5-Fu and other fluoropyrimidines (26, 41) had no significant difference in the two comparison groups in the current study.

We also used GSEA to dissect the pathways associated with 5-Fu resistance. Most of the pathways that have been shown to be involved in 5-Fu resistance were identified in the current research (**Figures 3E, F** **)** (42, 43). Pyrimidine metabolic resistance played a central role in 5-Fu resistance and was also identified here (**Figure S2A**) (42, 43). Additionally, other well-proven pathways regarding 5-Fu resistance discovered in the current analysis included mismatch repair, apoptosis, cell cycle, and mitochondria (oxidative phosphorylation) (**Figures S2B–O**). All enriched pathways were attached as supplementary materials (**Figures S3A–D**; **Tables S3A, B**). **Figures S3A–D** were deposited in the Mendeley Database (DOI: 10.17632/rnrmjkvjjc.2).

### Screening of Drug-Resistant Genes Associated With Prognosis in Colorectal Cancer Patients

**Figure 4:**
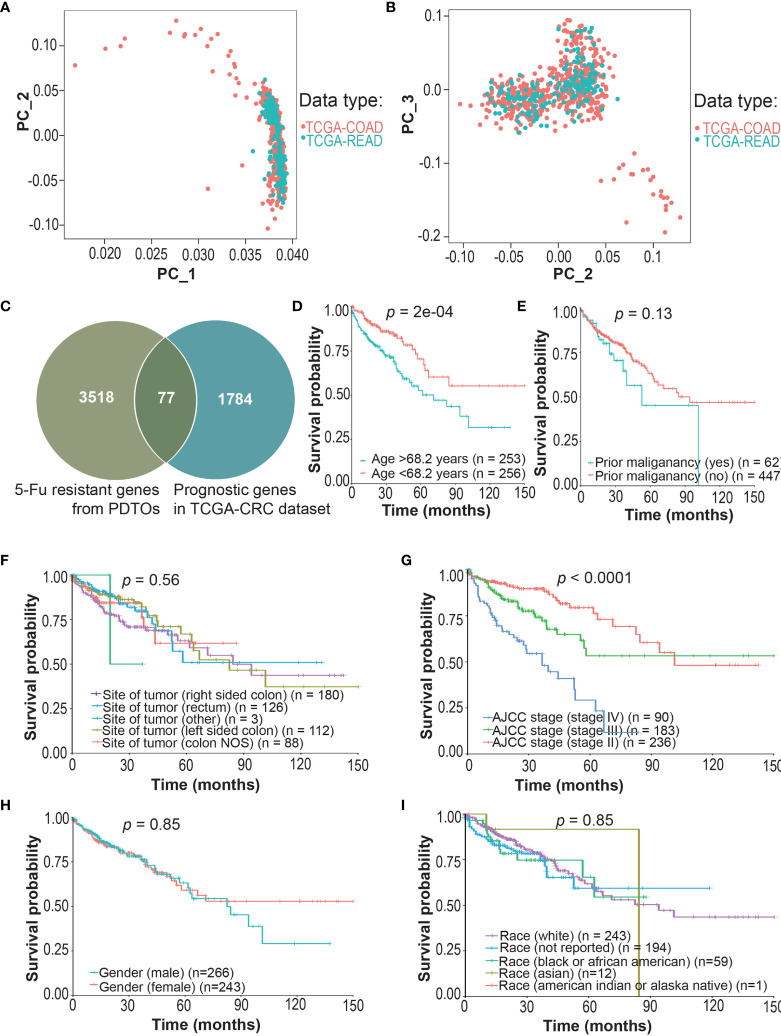
Screening of drug (5-Fu)-resistant genes (DRGs) associated with prognosis in colorectal cancer patients (CRCpts). **(A, B)** PCA for the gene expression data of TCGA-COAD and TCGA-READ cohorts. **(C)** Venn diagram of 5-Fu-resistant genes from patient-derived tumor organoids (PDTOs) and prognostic genes in TCGA-CRC dataset. **(D–I)** Univariate analysis for age/prior maliganancy/site of tumor/AJCC stage/gender/race and their correlation with clinical outcome [overall survival (OS)] in TCGA-CRC cohort. PC, principal components; TCGA, the cancer genome atlas; COAD, colon adenocarcinoma; READ, rectal adenocarcinoma; 5-Fu, 5-fluorouracil; PDTOs, patient-derived tumor organoids; CRC, colorectal cancer; NOS, not otherwise specified; AJCC, American Joint Committee on Cancer; DRGs, drug-resistant genes; CRCpts, colorectal cancer patients; PCA, principal components analysis; OS, overall survival.

First, we screened prognostic genes associated with survival of CRCpts in TCGA datasets. PCA showed that the gene expression data of TCGA-COAD and TCGA-READ could be integrated into a TCGA-CRC dataset for subsequent analysis (**Figures 4A, B** **)**. The results showed that the expression levels of 1,784 protein-coding genes were significantly associated with survival of CRCpts by using univariate Cox proportional hazards model analysis (**Figure 4C** and **Table S4**). Then, the DRGs were screened out among the 1,784 genes. There were 77 overlapped genes between 1,784 prognostic protein-coding genes and those DEGs associated with 5-Fu resistance derived from the CRC PDTOs (DRGs) (**Figure 4C** and **Table S5**).

Then, multivariate Cox analysis was performed to further screen candidate genes for the construction of DRSM. Variables with *P* value <0.25 in univariate test (44) or reported prognostic value were selected. Age (*P* = 2E-04), prior malignancy (*P* = 0.13), and site of tumor (45) and TNM stage (*P* < 0.0001) were included, but gender (*P* = 0.85) and race (*P* = 0.91) were excluded from the multivariate Cox model (**Figures 4D–I**). Multivariate Cox analysis disclosed that 46 of 77 DRGs’ expression levels were significantly correlated with the survival of CRCpts in TCGA-CRC dataset (**Table S6**). Expression data of these 46 genes would be used for the DRSM development next.

### Development of the Drug-Resistant Score Model by Using Drug-Resistant Genes Associated With 5-Fluorouracil Resistance

**Figure 5:**
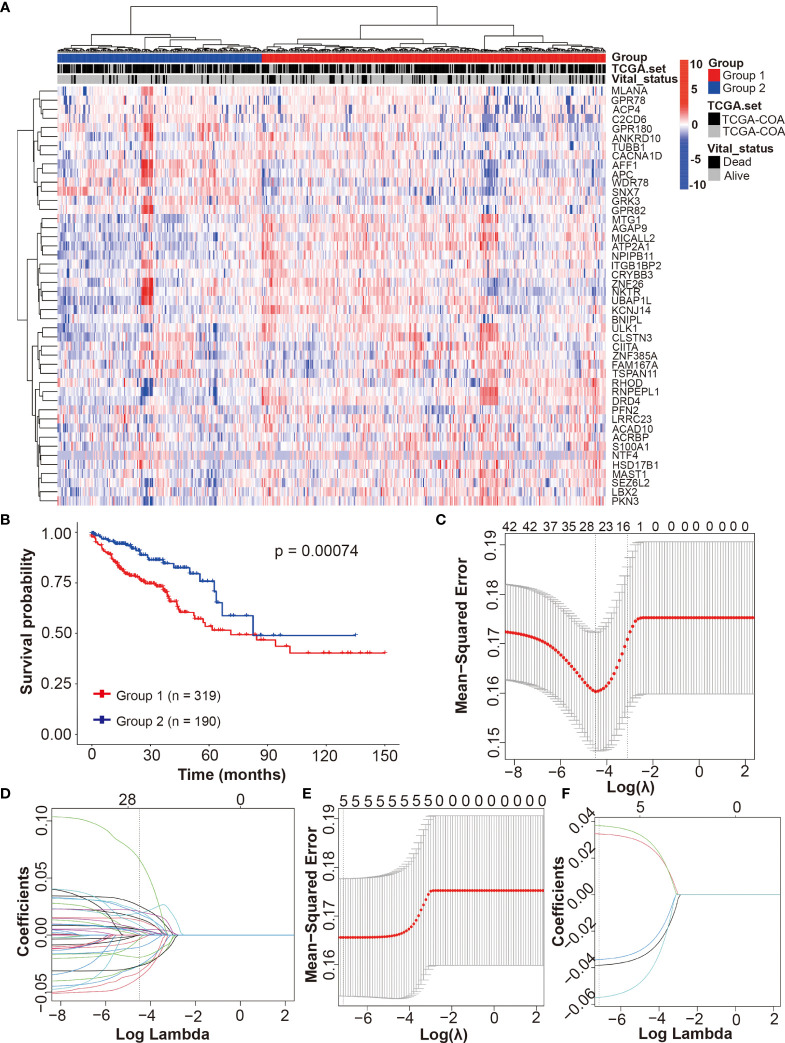
Development of the drug-resistant score model (DRSM) by using DRGs associated with 5-Fu resistance. **(A)** Unsupervised clustering of 698 tumor samples using the expression data of 46 DRGs in TCGA-CRC dataset revealed two molecular subtypes (group1, n = 319; group2, n = 190). **(B)** Univariate analysis for groups (1 or 2) and their correlation with clinical outcome [overall survival (OS)] in TCGA-CRC cohort. **(C)** Optimal parameter (lambda) selection in the LASSO model using 5-fold cross-validation *via* minimum criteria for 46 DRGs. **(D)** LASSO coefficient profiles of the 46 DRGs at the optimal lambda value selected using 5-fold cross-validation. **(E)** Optimal parameter (lambda) selection in the LASSO model using 5-fold cross-validation *via* minimum criteria for 5 DRGs after filtering using multivariate Cox analysis. **(F)** LASSO coefficient profiles of the 5 DRGs at the optimal lambda value selected using 5-fold cross-validation. TCGA, the cancer genome atlas; DRSM, drug-resistant score model; DRGs, drug-resistant genes; 5-Fu, 5-fluorouracil; CRC, colorectal cancer; OS, overall survival; LASSO, least absolute shrinkage and selection operator.

To evaluate the contribution of DRGs to CRCpts’ survival, we applied unsupervised clustering algorithms to group the expression data of 46 DRGs in TCGA-CRC dataset, and subsequently, the CRCpts were divided into Group1 (n = 319) and Group2 (n = 190) (**Figure 5A**). Univariate Cox analysis showed that patient survival of Group2 was significantly better than that of Group1 (*P* = 0.00074) (**Figure 5B**).

Then, LASSO regression analysis method and 5-fold cross-validation were used to develop the DRSM for CRCpts in TCGA-CRC dataset. After the best lambda value and coefficient of 46 DRGs were outputted (**Figures 5C, D** and **Table S7**), we obtained 26 genes with non-zero coefficients (**Table S8**). Next, these 26 genes were further filtered using multivariate Cox analysis in TCGA-CRC dataset. Five genes finally remained after the second filter: *CACNA1D* (*P* = 0.0019), *CIITA* (*P* = 0.00503), *PFN2* (*P* = 0.01176), *SEZ6L2* (*P* = 0.02853), and *WDR78* (*P* = 0.0305).

Afterward, the DRSM was established in TCGA-CRC dataset by LASSO regression analysis method and 5-fold cross-validation based on the five genes above (**Figures 5E, F** **)**. The result showed that coefficients of the five genes were all non-zero. The equation of DRS was finally derived: DRS = GEL (gene expression level) (*CACNA1D*) × -0.0563 + GEL (*CIITA*) × -0.0356 + GEL (*PFN2*) × 0.0332 + GEL (*SEZ6L2*) × 0.0378 + GEL (*WDR78*) × -0.0386.

**Figure 6:**
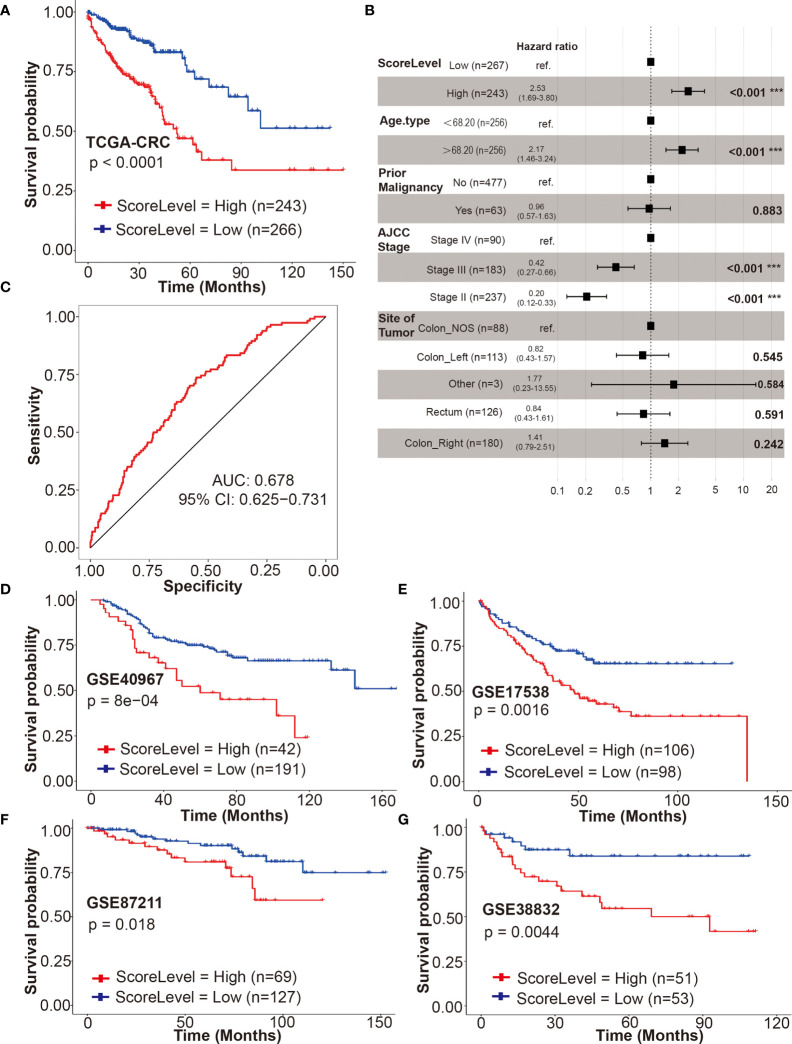
Validation of the DRSM. **(A)** Univariate analysis for drug-resistant score (DRS) (high or low) and its correlation with clinical outcome [overall survival (OS)] in TCGA-CRC cohort. **(B)** Multivariate analysis after adjustment revealed DRS, age, and AJCC stage were independent prognostic factors for OS in TCGA-CRC cohort, and prior malignancy and site of tumor lost their significance. **(C)** Receiver operating characteristic (ROC) curve (AUC = 0.99) indicated that DRSM had favorable prognosis predictive performance in TCGA-CRC dataset. AUC indicates area under the curve. **(D–G)** Univariate analysis for DRS (high or low) and its correlation with clinical outcome (OS or DSS) in GSE40967, GSE17538, GSE87211, and GSE38832 datasets, respectively. ***P < 0.001. TCGA, the cancer genome atlas; CRC, colorectal cancer; AJCC, American Joint Committee on Cancer; NOS, not otherwise specified; AUC, area under the curve; CI, confidence interval; GSE, gene expression omnibus series; DRSM, drug-resistant score model; DRS, drug resistant score; OS, overall survival; ROC, receiver operating characteristic; DSS, disease free survival.

In TCGA-CRC cohort, the univariate Cox regression model revealed that the DRS was associated with prognosis of CRC patients in terms of OS (*P* < 0.0001) (**Figure 6A**). Multivariate analysis after adjustment revealed that DRS (*P* < 0.001), age (*P* < 0.001), and American Joint Committee on Cancer (AJCC) stage (*P* < 0.001) were independent prognostic factors for OS in CRC patients, and prior malignancy (*P* = 0.883) and site of tumor (*P* > 0.2) lost their significance (**Figure 6B**). The result of receiver operating characteristic (ROC) curve (AUC = 0.99) indicated that our DRSM had a favorable prognosis predictive performance in TCGA-CRC dataset (**Figure 6C**).

### Validation of the Drug-Resistant Score Model

The prognosis predictive value of our DRSM was subsequently validated in four GSE datasets. Four GEO datasets [GSE39084 (n = 61), GSE71187 (n = 52), GSE12945 (n = 49), and GSE29623 (n = 37)] were excluded due to the small sample size. Three GEO datasets [GSE24551 (DFS), GSE33113 (RFS), and GSE14333 (DFS)] were not enrolled for validation because it was rare that endpoints such as disease free survival (DFS) progression free survival (PFS), or recurrence free survival (RFS) have been shown to be true surrogates for OS or disease free survival (DSS). Hence, 4 of 11 GEO datasets were finally selected for the validation of DRSM as follows: GSE40967 (n = 233, OS), GSE17538 (n = 204, OS), GSE87211 (n = 196, OS), and GSE38832 (n = 104, DSS).

Next, we validated the prognosis predictive value of our DRSM in GEO datasets. In all of the four enrolled GEO cohorts, the univariate Cox regression model indicated that the DRS was significantly associated with prognosis of CRC patients in terms of OS or DSS with the *P* values of 8e–04 (GSE40967), 0.0016 (GSE17538), 0.018 (GSE87211), and 0.0044 (GSE38832) (**Figures 6D–G**). Further multivariate analysis in three enrolled GEO cohorts (GSE40967, GSE17538, and GSE38832) also showed that DRS was an independent prognostic factor for OS or DSS in CRCpts (**Table S9**). In the GSE87211 cohort, the multivariate analysis was not performed because the event number was too limited (28 events out of 203 cases) and there were at least seven required variables for Cox regression (age, invasion depth, lymph node metastasis, metastasis, recurrence, KRAS mutations, and score level) (46). In GSE38832, there was no statistically significant difference (*P* = 0.093) between DRS-high and DRS-low CRCpts after multivariate Cox regression analysis probably due to the relatively small sample size (n = 104). Our validation results from the four GSE cohorts above elucidated that the DRSM based on five genes of chemosensitivity to 5-Fu developed from patient-derived organoids can predict the survival of CRCpts.

### Predictive Value of Drug-Resistant Score Model in Colorectal Cancer Patient Subgroups

**Figure 7:**
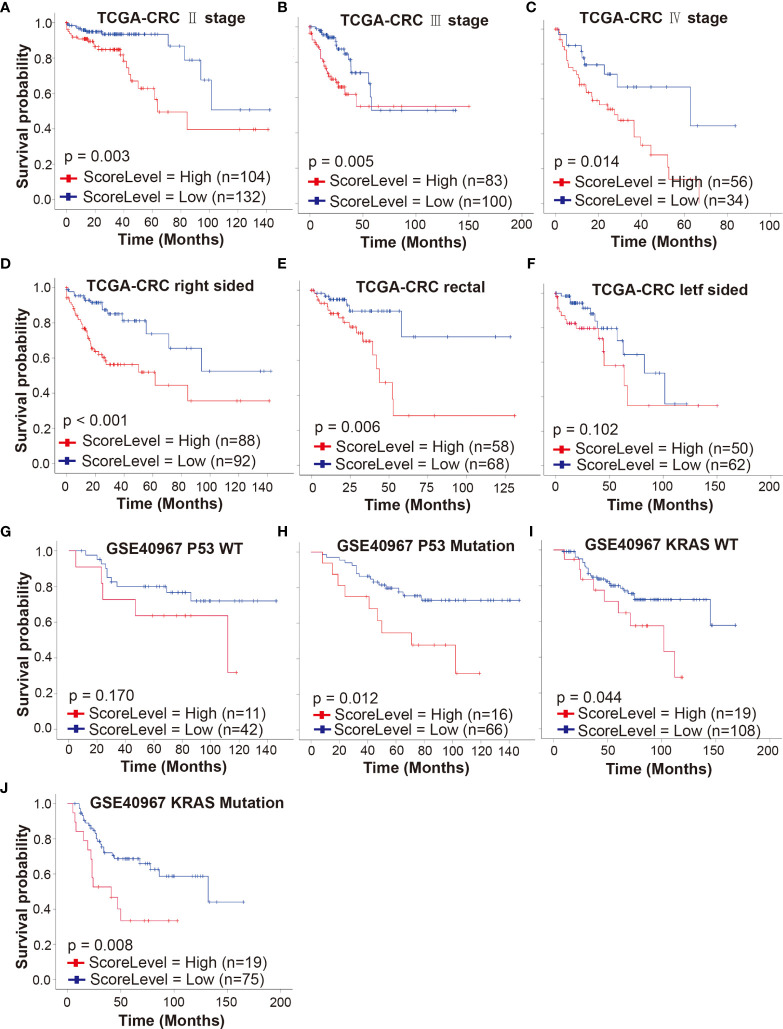
Predictive value of DRSM in CRCpt subgroups. **(A–C)** Univariate analysis for DRS (high or low) and its correlation with clinical outcomes (OS) of stage II, stage III, and stage IV CRCpts in TCGA-CRC cohort, respectively. **(D–F)** Univariate analysis for DRS (high or low) and its correlation with clinical outcomes (OS) of right-sided, rectal, and left-sided CRCpts in TCGA-CRC cohort, respectively. **(G, H)** Univariate analysis for DRS (high or low) and its correlation with clinical outcomes (OS) of CRCpts with wild-type and mutated P53 in GSE40967 cohort, respectively. **(I, J)** Univariate analysis for DRS (high or low) and its correlation with clinical outcomes (OS) of CRCpts with wild-type and mutated KRAS in GSE40967 cohort, respectively. TCGA, the cancer genome atlas; CRC, colorectal cancer; GSE, gene expression omnibus series; WT, wild type; DRSM, drug-resistant score model; CRCpts, colorectal cancer patients; DRS, drug resistant score; OS, overall survival.

To investigate whether our gene signature can serve as a powerful prognostic indicator in different stages of CRCpts, we performed a subset analysis based on AJCC staging system in TCGA-CRC cohort. The results from univariate and multivariate analyses showed that the DRSM could predict outcomes of stage II, III, and IV CRCpts, respectively (**Figures 7A–C** and **Table S10**). Embryological, biological, anatomical, and molecular features are different among right-sided, left-sided, and rectal CRC. Sidedness has an important role on several aspects of CRC (5). Next, we tested the prognostic value of DRSM according to tumor location in TCGA-CRC datasets. Univariate and multivariate survival analyses revealed that DRS-high CRCpts had worse survival than DRS-low CRCpts in right-sided colon cancer (*P* < 0.001, n = 180) and rectal cancer (*P* = 0.006, n = 126), but there was no statistical difference in left colon cancer due to the relatively small sample size (*P* = 0.102, n = 113) (**Figures 7D–F** and **Table S10**).

*TP53* and *KRAS* are second and third most frequently mutated genes among the non-hypermutated CRC tumors and contribute to colorectal carcinogenesis. *KRAS* mutations predict poor prognosis in CRC (47, 48). Univariate and multivariate survival analyses in GSE40967 cohort showed that the DRS-high CRCpts had worse survival than DRS-low CRCpts with *P53* mutations (*P* = 0.012, n = 82) (**Figure 7G** and **Table S11**). There was no statistically significant difference between DRS-high and DRS-low CRCpts with wild-type *P53* probably because of the small sample size (*P* = 0.170, n = 53) (**Figure 7H** and **Table S11**). Univariate and multivariate Cox analyses further exhibited that our DRSM could serve as an independent predictor of both *KRAS*-mutated and wild-type CRCpts’ survival in the GSE40967 dataset (*P* = 0.008, n = 94; *P* =0.044, n = 127) (**Figures 7I, J** and **Table S11**). We did not perform subgroup analysis according to CpG island methylator phenotype (CIMP), CIN, *BRAF* mutation, and subtypes from the French national Cartes d’Identité des Tumeurs (CIT) program in the GSE40967 cohort because of small sample size of subgroups. There were no appropriate additional variables for subgroup analysis in the GSE17538, GSE87211, and GSE38832 cohorts.

### Functional Enrichment Analyses Between DRS-High and DRS-Low Patients

**Figure 8:**
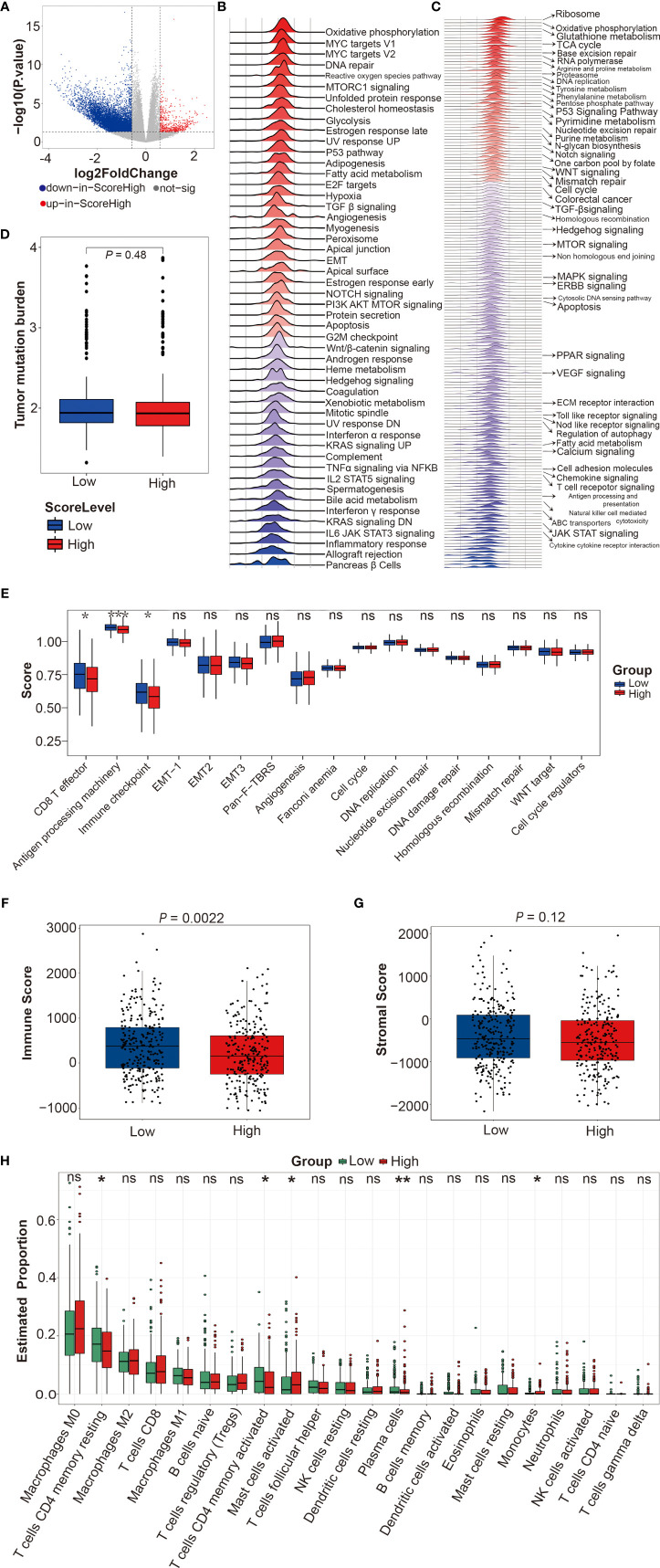
Functional enrichment analyses between DRS-high and DRS-low patients in TCGA-CRC cohort. **(A)** Volcano plot for differentially expressed genes of CRC tumors between DRS-high and DRS-low CRCpts in TCGA-CRC cohort. **(B, C)** Gene set enrichment analysis (GSEA) using the hallmark and KEGG gene sets to dissect the pathways associated with DRS in TCGA-CRC cohort. **(D)** Box plots of tumor mutational burden (TMB) by DRS (low or high). **(E)** Box plots of immune response-related pathways by DRS (low or high). **(F, G)** Box plots of immune score and stromal score by DRS (low or high). **(H)** Box plots of immune cell proportion by DRS (low or high). Within each box, the horizontal black center line denotes the median value (50th percentile), while the box contains the 25th to 75th percentiles of each group’s distribution of values. The black whiskers mark the 5th and 95th percentiles, and values beyond these upper and lower bounds are considered outliers. *P < 0.05; **P < 0.01; ***P < 0.001; ns, not significant. sig, significant; DRS, drug resistant score; TCGA, the cancer genome atlas; CRC, colorectal cancer; CRCpts, colorectal cancer patients; GSEA, gene set enrichment analysis; KEGG, kyoto encyclopedia of genes and genomes; TMB, tumor mutational burden.

Finally, we used DEGs between DRS-high and DRS-low patients in TCGA-CRC cohort to dissect the difference of molecular pathways, tumor mutational burden (TMB), immune response-related pathways, immune score, stromal score, and immune cell proportion (**Figure 8A** and **Table S12**). GSEA method was employed to determine the upregulated and downregulated molecular pathways in DRS-high CRCpts based on the kyoto encyclopedia of genes and genomes (KEGG) and Hallmark gene sets. The MYC targets, reactive oxygen species pathway, base excision repair, citrate cycle, and tricarboxylic acid (TCA) cycle were upregulated, while KRAS signaling, ABC transporters, calcium signaling pathway, and chemokine signaling pathway were downregulated in DRS-high CRCpts (**Figures 8B, C** **)**. Detailed information about upregulated and downregulated molecular pathways was listed in supplementary materials (**Figures S4A, B** and **Table S13**). **Figures S4A, B** were deposited in the Mendeley Database (DOI: 10.17632/rnrmjkvjjc.2).

TMB is a measure of the amount of mutations carried by tumor cells. TMB-low is associated with poor prognosis in CRCpts treated with adjuvant 5-Fu-based chemotherapy (49). However, there was no significant difference in TMB between DRS-high and DRS-low CRCpts (**Figure 8D**). Next, we further explored the differences in immune response-related pathways, immune score, stromal score, and immune cell proportion between DRS-high and DRS-low CRCpts. Our results found that CD8 T effector, antigen processing machinery, and immune checkpoint pathways were significantly downregulated in DRS-high CRCpts (**Figure 8E**). Immune score was significantly lower in DRS-high CRCpts (*P* = 0.0022) (**Figure 8F**). There was no significant difference in stromal score between DRS-high and DRS-low CRCpts (*P* = 0.12) (**Figure 8G**). For immune cell proportion, T cell CD4 memory resting, T cell CD4 memory activated, and plasma cells were lower and mast cell activated and monocytes were higher in DRS-high CRCpts (**Figure 8H**).

## Discussion

In this study, we investigated whether DRGs developed from PDTOs could be used to faithfully identify robust drug response biomarkers. The 5-Fu-resistant genes were established by analysis of RNA sequencing data from CRCOs and were employed to generate the DRSM using LASSO regression analysis in TCGA and GSE CRC datasets. Indeed, we found that gene signatures of 5-Fu resistance derived from CRCOs could predict the survival of CRCpts. Our results suggested that genetic characteristics of drug resistance in PDTOs could improve the drug response prediction for cancer patients.

Until now, genome-wide mRNA expression levels in CRC have been obtained in lots of studies by using large-scale genomic profiling technology. Many gene expression signatures for survival prediction of CRCpts also have been developed (50–52), but none was routinely used in the clinic. A systematic review including 31 gene signatures concluded that although the published signatures showed significant statistical correlation with prognosis, their capacity to accurately categorize independent samples into low-risk and high-risk subgroups remained limited (52). Consistent with the conclusion above, the results of the current study in validation cohorts (GSE datasets) demonstrated that the prediction power of our gene signatures was moderate, with AUC ranging from 0.557 (95% CI: 0.476−0.639) to 0.672 (95% CI: 0.549−0.794). Strong prediction power is necessary for gene signatures to be used clinically, even when patients’ survival differences exist. Thus, further well-designed research with a large sample size is needed for developing gene signatures with higher predictive accuracy in CRCpts.

5-Fu-resistant genes generated from CRC cell lines have been used to construct gene signatures to predict survival of CRCpts (34, 53, 54). Considering the advantage of PDTOs over cell lines (8, 9), gene signatures derived from PDTOs may exhibit better predictive power. Kong et al. recently reported that biomarkers that were identified by network-based machine learning using 5-Fu pharmacogenomic data generated from 19 3D organoid lines accurately predicted the drug responses of 114 CRCpts (14, 55). In the current study, the 5-Fu-resistant genes were obtained from pharmacogenomic and expression data of 41 CRCOs. In addition to comparing 5-Fu-sensitive and -resistant CRCOs, we analyzed the gene expression data of CRCOs before 5-Fu treatment and surviving CRCOs after 5-Fu treatment to generate 5-Fu-resistant genes. It is worth noting that 5-Fu is generally used in combination with oxaliplatin for CRCpts clinically. The treatments of CRCpts, especially the drug information, were often unavailable in TCGA-CRC and GSE datasets used in the current study. Since 5-Fu is the critical composition of CRC treatments and used in a vast majority of CRCpts, we only employed the sensitivity data of CRCOs to 5-Fu for the development of the DRSM.

Of note, we used organoid size change (d24/d0) after a single dose (10 μM) of 5-Fu treatment to evaluate the drug sensitivity of CRCOs in the current study. IC50 after 6 days of drug treatment was regularly employed in other studies about drug sensitivity tests of cancer organoids (10, 14, 22, 23). The former method was selected in our study according to a research about testing the response of rectal cancer organoids (RCOs) to drugs and irradiation (11). Yao et al. (11) tested the response of 80 RCOs to 5-Fu (10 μM), irinotecan (CPT-11), and irradiation by using organoid size change (d24/d0) to evaluate the drug sensitivity of RCOs and found that the organoid data were highly matched to clinical outcomes of rectal cancer patients (RCpts). In that study, outcomes of RCpts were accessed by pathologic tumor regression grade (TRG) of surgical specimens after neoadjuvant chemoradiation (11). Considering the sufficient sample size and reliable evaluation methods of clinical outcomes (11), organoid size change (d24/d0) is a valid parameter for testing the response of cancer organoids to treatments. This method is also as effective as CellTiter-Glo 3D cell viability assay (11) and is more economical and easier to use. In addition, we chose 36.4% as the cutoff of organoid size change (d24/d0) according to a previous study (11). This cutoff was derived based on the fact that the primary tumors of patients with TRG = 0 or 1 were considered to be sensitive to treatments and other patients with TRG = 2 or 3 were resistant (11). The 5-year recurrence-free survival rates were 98% (TRG = 0), 90% (TRG =1), 73% (TRG = 2), and 68% (TRG = 3) (56). By using this cutoff in the current study, more than half of PTDOs are considered to be resistant to 5-Fu. In fact, the patients with TRG = 2 can also benefit from neoadjuvant chemoradiation. Given that we aimed to develop a molecular biomarker of CRCpts’ survival, this cutoff was exactly appropriate for the current study.

Our DRSM consisted of five genes, namely, *CACNA1D*, *CIITA*, *PFN2*, *SEZ6L2*, and *WDR78*. CACNA1D encodes the α 1D subunit of the L-type calcium channel and is engaged in various calcium-dependent processes, including neurotransmitter or hormone release, muscle contraction, and gene expression. *CACNA1D* showed significant correlations with chemosensitivity for mitozolamide, cyclodisone, and deoxydoxorubicin (57). *CACNA1D* has been also enrolled in the gene signatures for predicting the benefit of 5-Fu-based chemotherapy (58, 59). *CIITA* is a non-DNA-binding coactivator of major histocompatibility complex (MHC) class II molecules whose high expression is usually associated with enhanced involvement of CD4+ lymphocytes in tumor suppression and a better prognosis (60). *PFN2* is an actin cytoskeleton regulator and serves an important role in cell motility. The results from Kim et al. (61) suggested that *PFN2* promoted the migration, invasion, and stemness of HT29 human CRC stem cells. *SEZ6L2* is a type 1 transmembrane protein and belongs to the seizure‐related gene 6 (*SEZ6*) family. Upregulation of *SEZ6L2* correlates with poor prognosis for CRCpts, and S*EZ6L2* knockdown can impair tumor growth by promoting caspase‐dependent apoptosis in CRC (62). *WDR78* is essential for ciliary beating and axonemal dyneins. Studies showed that *WDR78* has been enrolled in the molecular signatures for predicting the prognosis of CRCpts (63, 64). In further studies, we will explore the specific roles and mechanisms of the five genes in 5-Fu resistance in CRC.

The current research, however, is subjected to several limitations. The first is the limited sample size of CRCOs. We tested the response of 41 CRCO lines to 5-Fu to generate 5-Fu-resistant genes. The sample size needs to be expanded in further research. The second limitation concerns that only 5-Fu-resistant genes were derived, while resistant genes of other clinically used drugs (oxaliplatin, CPT-11, et al.) for CRCpts were not. In previous research, 5-Fu-based chemotherapy improves the survival of resected stage III, a subset of stage II and metastatic CRCpts (5, 65). Given the fact that drugs other than 5-Fu used for CRCpts in TCGA and GSE datasets were unable to be confirmed, we just utilized 5-Fu-resistant genes to construct the prediction model. Oxaliplatin- and CPT-11-resistant genes will be incorporated in the model in further study using our own independent CRCpt cohort. In addition, as with majority of similar studies, the design of the current study is retrospective. Our prediction model needed further validation in prospective clinical studies.

In the current era of precision and personalized cancer medicine, molecular biomarkers enabling selection of the appropriate treatments for specific patients are of great importance (66–68). Cancer organoid technology, together with molecular biomarkers, holds promise for individualizing cancer treatment. We here provide suggestions that gene signatures of drug resistance developed from CRC PTDOs have the potential to be possible candidates of such molecular biomarkers.

## Conclusions

Taken together, the DRSM developed in the current study by using 5-Fu-resistant genes derived from CRCOs can predict the survival of CRCpts in TCGA and GSE CRC datasets. This gene signature may be useful in tailoring therapeutic regimens and acts as a supplement of PDTO-guided personalized treatment for CRCpts. Further study with a large sample size and even a prospective design is needed.

## Data Availability Statement

The RNA sequencing data (raw data) of colorectal cancer organoids presented in the study are deposited in the Sequence Read Archive (SRA) of National Center for Biotechnology Information (NCBI), accession number PRJNA813221. The **Figures S3** and **S4** presented in the study are deposited in the Mendeley Database, DOI 10.17632/rnrmjkvjjc.2. Additional datasets and materials and associated protocols are available upon request from the corresponding author (YY and WZL) to comply with institutional ethics regulation.

## Ethics Statement

The studies involving human participants were reviewed and approved by Ethics Committee of Tongji Medical College, Huazhong University of Science and Technology. The patients/participants provided their written informed consent to participate in this study.

## Author Contributions

LFC and WZL designed the current study and supervised the project. YY also supervised the project. LFC and BT executed most of the experiments and bioinformatics analysis. WZL was responsible for CRC tumor sample collection. LFC and BT wrote the article. WZL and YY provided support for research funding. BT, WL, HTL, and WZL revised the article. All authors read and approved the final article.

## Funding

This work was supported by the National Natural Science Foundation of China (No. 81873440).

## Conflict of Interest

The authors declare that the research was conducted in the absence of any commercial or financial relationships that could be construed as a potential conflict of interest.

## Publisher’s Note

All claims expressed in this article are solely those of the authors and do not necessarily represent those of their affiliated organizations, or those of the publisher, the editors and the reviewers. Any product that may be evaluated in this article, or claim that may be made by its manufacturer, is not guaranteed or endorsed by the publisher.

## Supplementary Material

Figures S3 and S4 are available here: DOI 10.17632/rnrmjkvjjc.2.
