# Supplementary material for: Molecular Biomarker of Drug Resistance Developed From Patient-Derived Organoids Predicts Survival of Colorectal Cancer Patients
Source: Front Oncol. 2022 Mar 29;12:855674. doi: 10.3389/fonc.2022.855674 (PMC9004628; doi:10.3389/fonc.2022.855674)
Supplement: Supplementary file 1 [file DataSheet_1.pdf]

Lifeng Chen, et al. Figure S1A

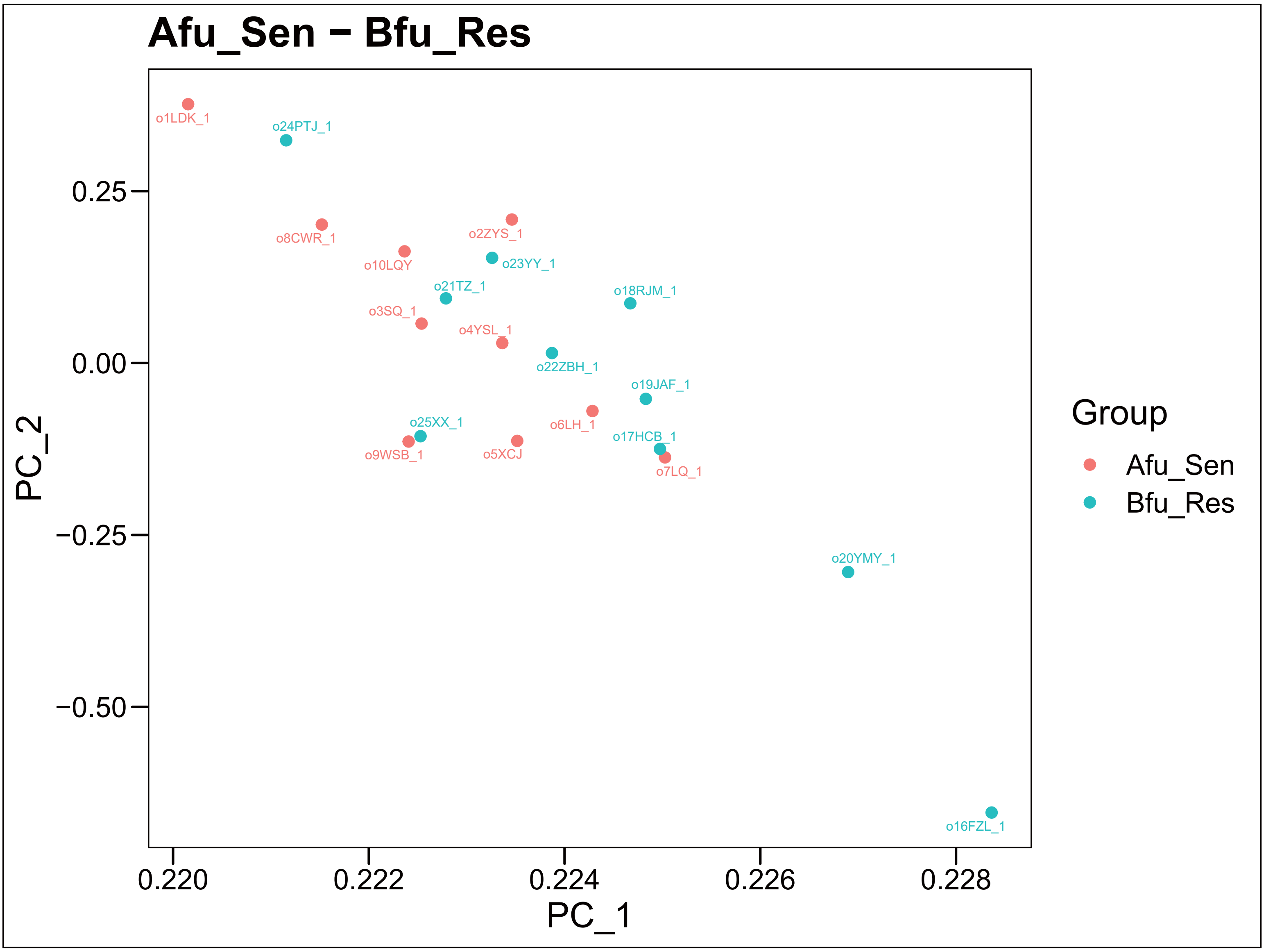

Lifeng Chen, et al. Figure S1B

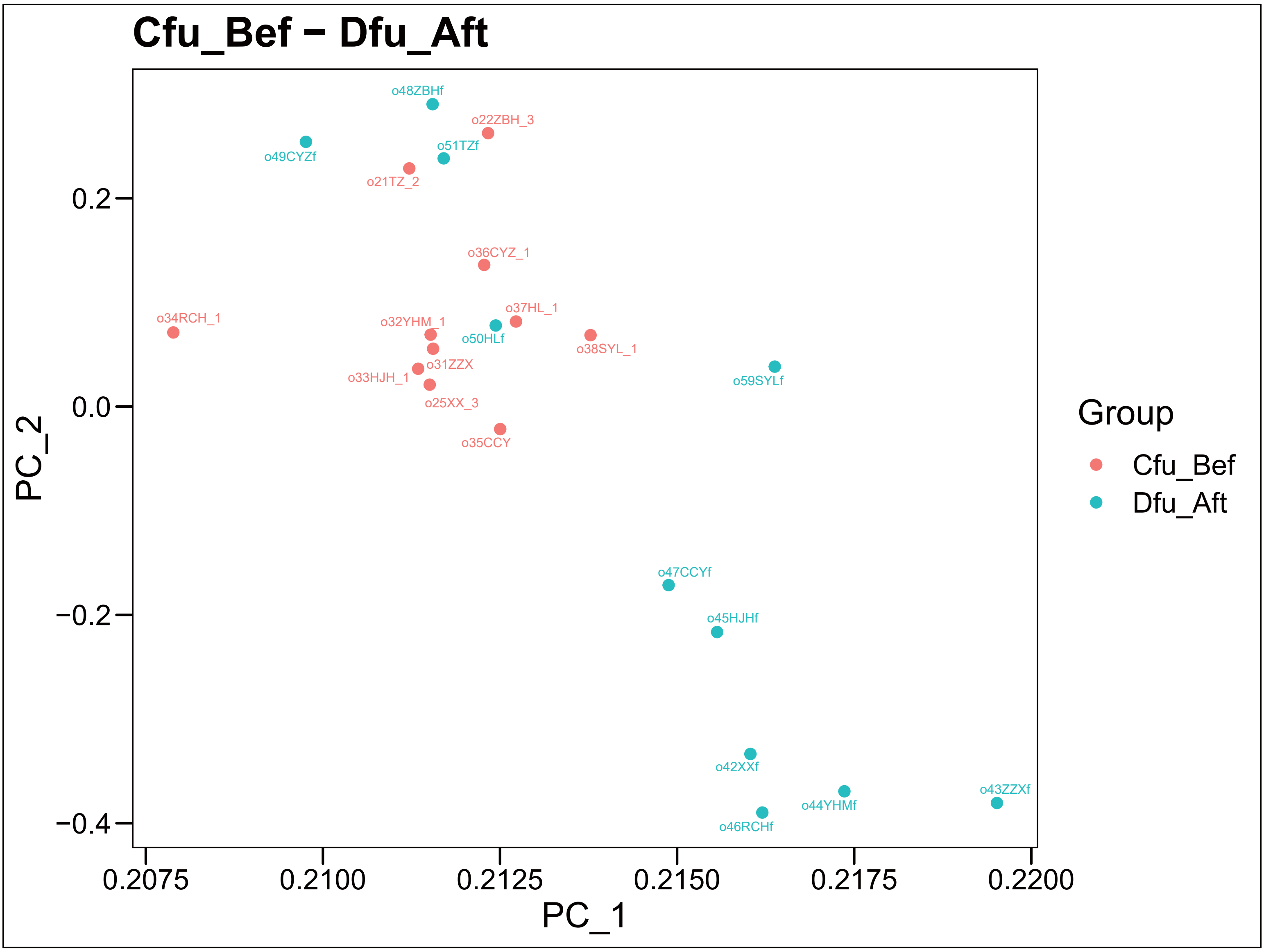

Figure S1. Principal component analysis (PCA) showed high degree of similarity between group A/B and C/D, respectively. Group A: 5-Fu sensitive untreated CRCOs; Group B: 5-Fu resistant untreated CRCOs; Group C: CRCOs before 5-Fu treatment; Group D: surviving CRCOs after 5-Fu treatment.
