# Supplementary material for: Molecular Biomarker of Drug Resistance Developed From Patient-Derived Organoids Predicts Survival of Colorectal Cancer Patients
Source: Front Oncol. 2022 Mar 29;12:855674. doi: 10.3389/fonc.2022.855674 (PMC9004628; doi:10.3389/fonc.2022.855674)
Supplement: Supplementary file 2 [file DataSheet_2.pdf]

Lifeng Chen, et al. Figure S2

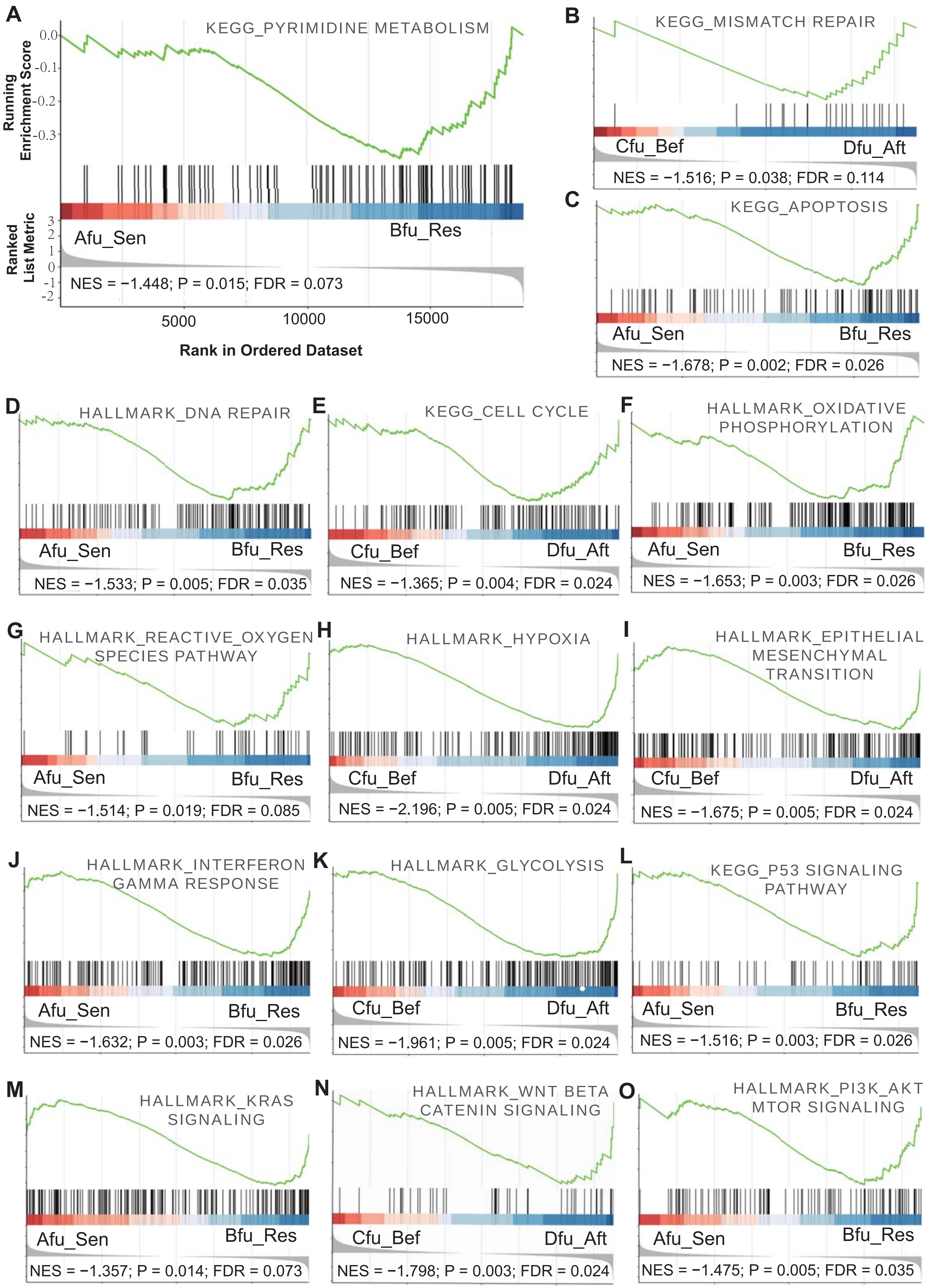

Fig. S2 Enrichment plots of GSEA for well-proven pathways regarding 5-Fu resistance discovered in current analysis. **a** Enrichment plot for pyrimidine metabolic pathway. **b-o** Enrichment plots for mismatch repair, apoptosis, DNA repair, cell cycle, oxidative phosphorylation, reactive oxygen species pathway, hypoxia, epithelial mesenchymal transition, interferon gamma response, glycolysis, P53 signaling, KRAS signaling, WNT beta catenin signaling and PI3K-AKT-MTOR signaling pathways.
