# Supplementary material for: Molecular Biomarker of Drug Resistance Developed From Patient-Derived Organoids Predicts Survival of Colorectal Cancer Patients
Source: Front Oncol. 2022 Mar 29;12:855674. doi: 10.3389/fonc.2022.855674 (PMC9004628; doi:10.3389/fonc.2022.855674)
Supplement: Supplementary file 3 [file DataSheet_3.zip › Data sheet 3/Table S1.docx]

**Table S1**. Patient characteristics associated to PTDOs generation and organoid sensitivity to 5-FU

| **Characteristics** | **PTDOs generation** | |  |  | **Organoid sensitivity to 5-FU** | |  |
| --- | --- | --- | --- | --- | --- | --- | --- |
|  | Yes (n) | No (n) | *P* |  | Sensitive (n) | Resistance (n) | *P* |
| **Sex** |  |  | **0.69** |  |  |  | **0.86** |
| Male | 30 | 6 |  |  | 10 | 20 |  |
| Female | 11 | 3 |  |  | 4 | 7 |  |
| **Age** |  |  | **0.23** |  |  |  | **0.45** |
| ≥53 years | 23 | 7 |  |  | 9 | 14 |  |
| ＜53 years | 18 | 2 |  |  | 5 | 13 |  |
| **T stage** |  |  | **0.39** |  |  |  |  |
| T2 | 7 | 0 |  |  | 3 | 4 | **0.71** |
| T3 | 29 | 8 |  |  | 10 | 19 |  |
| T4 | 5 | 1 |  |  | 1 | 4 |  |
| **N stage** |  |  | **0.91** |  |  |  | **0.76** |
| N0 | 4 | 1 |  |  | 2 | 2 |  |
| N1 | 7 | 1 |  |  | 2 | 5 |  |
| N2 | 30 | 7 |  |  | 10 | 20 |  |
