# Supplementary material for: Molecular Biomarker of Drug Resistance Developed From Patient-Derived Organoids Predicts Survival of Colorectal Cancer Patients
Source: Front Oncol. 2022 Mar 29;12:855674. doi: 10.3389/fonc.2022.855674 (PMC9004628; doi:10.3389/fonc.2022.855674)
Supplement: Supplementary file 3 [file DataSheet_3.zip › Data sheet 3/Table S10.docx]

Table S10. Multivariate analysis in subgroups (TCGA-CRC cohort)

| **Variables** | **P values** | **Hazard ratio (95%CI)** |
| --- | --- | --- |
| **TCGA-CRC cohort (Ⅱ stage)** | | |
| Age (<68.2 years) | 0.003 | 0.223 (0.083 - 0.598) |
| Prior maglignancy (no/yes) | 0.940 | 1.043 (0.352 - 3.088) |
| Site | 0.990 | - |
| Score level (low/high) | 0.007 | 0.355 (0.168 – 0.751) |
| **TCGA-CRC cohort (Ⅲ stage)** | | |
| Age (<68.2 years) | 0.023 | 0.450 (0.226 – 0.895) |
| Prior maglignancy (no/yes) | 0.332 | 0.660 (0.284 – 1.530) |
| Site | 0.750 | - |
| Score level (low/high) | 0.011 | 0.427 (0.222 – 0.823) |
| **TCGA-CRC cohort (Ⅳ stage)** | | |
| Age (<68.2 years) | 0.159 | 0.611 (0.307 – 1.213) |
| Prior maglignancy (no/yes) | 0.223 | 1.762 (0.709 – 4.380) |
| Site | 0.001 | - |
| Score level (low/high) | 0.010 | 0.353 (0.160 – 0.779) |
| **TCGA-CRC cohort (right-sided)** | | |
| Age (<68.2 years) | 0.014 | 0.445 (0.233 – 0.852) |
| Prior maglignancy (no/yes) | 0.878 | 1.063 (0.489 – 2.311) |
| Stage (Ⅱ/Ⅳ) | 0.000 | 0.130 (0.062 – 0.271) |
| Stage (Ⅲ/Ⅳ) | 0.000 | 0.263 (0.131 – 0.529) |
| Score level (low/high) | 0.001 | 0.331 (0.176 – 0.621) |
| **TCGA-CRC cohort (rectal)** | | |
| Age (<68.2 years) | 0.101 | 0.494 (0.212 – 1.148) |
| Prior maglignancy (no/yes) | 0.429 | 1.817 (0.414 – 7.978) |
| Stage (Ⅱ/Ⅳ) | 0.016 | 0.227 (0.068 – 0.756) |
| Stage (Ⅲ/Ⅳ) | 0.115 | 0.469 (0.182 – 1.204) |
| Score level (low/high) | 0.047 | 0.372 (0.140 – 0.986) |
| **TCGA-CRC cohort (left-sided)** | | |
| Age (<68.2 years) | 0.188 | 0.543 (0.219 – 1.347) |
| Prior maglignancy (no/yes) | 0.550 | 0.702 (0.220 – 2.243) |
| Stage (Ⅱ/Ⅳ) | 0.054 | 0.336 (0.111 – 1.020) |
| Stage (Ⅲ/Ⅳ) | 0.678 | 0.812 (0.303 – 2.174) |
| Score level (low/high) | 0.152 | 0.546 (0.239 – 1.250) |
