# Supplementary material for: Molecular Biomarker of Drug Resistance Developed From Patient-Derived Organoids Predicts Survival of Colorectal Cancer Patients
Source: Front Oncol. 2022 Mar 29;12:855674. doi: 10.3389/fonc.2022.855674 (PMC9004628; doi:10.3389/fonc.2022.855674)
Supplement: Supplementary file 3 [file DataSheet_3.zip › Data sheet 3/Table S11.docx]

Table S11. Multivariate analysis in subgroups (GSE40967 cohort)

| **Variables** | **P values** | **Hazard ratio (95%CI)** |
| --- | --- | --- |
| **P53 mutations** | | |
| Age (<64.6 years) | 0.659 | 0.835 (0.375 – 1.858) |
| Site (proximal/distal) | 0.915 | 0.945 (0.337 – 2.650) |
| Stage (Ⅱ/Ⅳ) | 0.001 | 0.070 (0.015 – 0.321) |
| Stage (Ⅲ/Ⅳ) | 0.000 | 0.048 (0.011 – 0.202) |
| Score level (low/high) | 0.006 | 0.268 (0.104 – 0.687) |
| **P53 wild type** | | |
| Age (<64.6 years) | 0.908 | 0.939 (0.320 – 2.753) |
| Site (proximal/distal) | 0.588 | 1.342 (0.462 – 3.895) |
| Stage (Ⅱ/Ⅳ) | 0.008 | 0.087 (0.014 – 0.528) |
| Stage (Ⅲ/Ⅳ) | 0.007 | 0.089 (0.015 – 0.513) |
| Score level (low/high) | 0.127 | 0.388 (0.115 – 1.311) |
| **KRAS mutations** | | |
| Age (<64.6 years) | 0.733 | 1.131 (0.557 – 2.297) |
| Site (proximal/distal) | 0.865 | 0.942 (0.470 – 1.887) |
| Stage (Ⅱ/Ⅳ) | 0.000 | 0.148 (0.054 – 0.403) |
| Stage (Ⅲ/Ⅳ) | 0.000 | 0.066 (0.023 – 0.188) |
| Score level (low/high) | 0.000 | 0.202 (0.090 – 0.454) |
| **KRAS wile type** | | |
| Age (<64.6 years) | 0.174 | 0.601 (0.289 – 1.252) |
| Site (proximal/distal) | 0.558 | 1.309 (0.532 – 3.219) |
| Stage (Ⅱ/Ⅳ) | 0.000 | 0.059 (0.019 – 0.180) |
| Stage (Ⅲ/Ⅳ) | 0.000 | 0.066 (0.024 – 0.179) |
| Score level (low/high) | 0.014 | 0.343 (0.146 – 0.803) |
