# Supplementary material for: Molecular Biomarker of Drug Resistance Developed From Patient-Derived Organoids Predicts Survival of Colorectal Cancer Patients
Source: Front Oncol. 2022 Mar 29;12:855674. doi: 10.3389/fonc.2022.855674 (PMC9004628; doi:10.3389/fonc.2022.855674)
Supplement: Supplementary file 3 [file DataSheet_3.zip › Data sheet 3/Table S9.docx]

Table S9. Multivariate analysis for validation of DRSM in GSE cohorts.

| **Variables** | **P values** | **Hazard ratio (95%CI)** |
| --- | --- | --- |
| **GSE40967 (n = 233; events number = 75)** | | |
| Age (<64.6 years) | 0.168 | 0.711 (0.438 – 1.154) |
| Site (proximal/distal) | 0.077 | 1.682 (0.945 – 2.995) |
| Stage (Ⅱ/Ⅳ) | 0.000 | 0.125 (0.055 – 0.285) |
| Stage (Ⅲ/Ⅳ) | 0.000 | 0.066 (0.028 – 0.155) |
| P53 mutation | 0.376 | - |
| KRAS mutation | 0.064 | - |
| BRAF mutation | 0.171 | - |
| CIT molecular subtype | 0.520 | - |
| Score Level (low/high) | 0.000 | 0.286 (0.156 – 0.524) |
| **GSE17538 (n = 204; events number = 89)** | | |
| Age (<65 years) | 0.045 | 0.633 (0.404 – 0.990) |
| Grade | 0.038 | - |
| Stage (Ⅱ/Ⅳ) | 0.000 | 0.166 (0.089 – 0.311) |
| Stage (Ⅲ/Ⅳ) | 0.000 | 0.251 (0.151 – 0.417) |
| Score Level (low/high) | 0.041 | 0.616 (0.387 – 0.979) |
| **GSE38832 (n = 104; events number = 28)** | | |
| Stage (stage Ⅱ + Ⅲ/stage Ⅳ) | 0.000 | 0.027 (0.008 – 0.090) |
| Score Level (low/high) | 0.093 | 0.474 (0.199 – 1.131) |
